# An Unusual Presentation of Renal Failure: A Case of Myeloperoxidase-Antineutrophil Cytoplasmic Antibody (MPO-ANCA) Vasculitis

**DOI:** 10.7759/cureus.52290

**Published:** 2024-01-15

**Authors:** Manasa Srinivasa Murthy, Dhanush Hoskere, Harish Veerapalli, Eiton Arroyo Rodriguez

**Affiliations:** 1 Internal Medicine, Bayonne Medical Center, Bayonne, USA; 2 Gastroenterology, Bayonne Medical Center, Bayonne, USA

**Keywords:** rapidly progressive renal failure, chronic kidney disease (ckd), chronic kidney disease, small vessel vasculitis, mpo-anca, mpo-anca vasculitis

## Abstract

Myeloperoxidase-antineutrophil cytoplasmic antibody (MPO-ANCA) vasculitis manifests as a neutrophilic inflammation impacting small vessels across multiple organs, notably the lungs, kidneys, and skin. We present a unique case of MPO-ANCA vasculitis in a 77-year-old female characterized by glomerulosclerosis, rapidly progressive renal failure necessitating hemodialysis (HD), bullous skin lesions, and hypoxic respiratory failure. The patient, who had a history of type 2 diabetes, presented with progressive dyspnea, hypoxia, and acute kidney injury superimposed on chronic kidney disease (CKD) progressing to renal failure requiring dialysis. A renal biopsy highlighted globally sclerosed glomeruli, interstitial fibrosis, and tubular atrophy, along with increased immunoglobulin M (IgM) deposits on immunofluorescence, differing from typical findings. Prompt initiation of prednisone led to respiratory and cutaneous improvement; however, despite therapy, extensive renal damage led to the permanent requirement of dialysis.

MPO vasculitis primarily targets small vessels, frequently affecting kidneys, with only a subset of patients progressing rapidly to end-stage renal failure necessitating HD, as observed in our case. Contrary to classical histopathological patterns, our patient exhibited augmented IgM deposits. Left untreated, MPO vasculitis with renal involvement poses a mortality risk of up to 90%, underscoring the significance of prompt detection and corticosteroid intervention to avert renal failure and improve patient outcomes. Early recognition and timely treatment are pivotal in mitigating the dire consequences of this condition, emphasizing the importance of considering MPO vasculitis in patients with rapidly deteriorating renal function.

## Introduction

MPO-ANCA vasculitis is characterized by neutrophilic inflammation impacting small vessels, commonly affecting the lungs, kidneys, and skin [1]. While renal involvement is frequent in these patients, cases leading to rapidly progressing renal failure necessitating hemodialysis (HD) are rare, making the case we present here noteworthy.

## Case presentation

A 77-year-old female with a past medical history of diabetes mellitus type 2 presented with progressive shortness of breath and anuria. Upon admission, the patient developed hypoxia with an oxygen saturation of 89%, and chest CT showed bilateral lower lobe pulmonary edema. The echocardiogram showed preserved left ventricular ejection fraction and no signs of diastolic dysfunction. Initial labs were significant for acute kidney injury on chronic kidney disease (CKD) with the glomerular filtration rate reduced to 19 from a baseline of 30. Urinalysis was positive for protein, white blood cells, and red blood cells.

The course was complicated by new-onset anuria requiring HD and the development of purplish bullous crusty lesions on the forehead. Workup of worsening renal function showed anti-nuclear antibody positive at 1:640 with a homogenous pattern, perinuclear antineutrophil cytoplasmic antibodies (p-ANCA) titer of more than 1:640 on immunofluorescence assay, MPO antibody of more than 800 on ELISA, and low complement component 3 (C3) at 72. Renal biopsy showed that 73% of glomeruli were globally sclerosed with additional findings of interstitial fibrosis and tubular atrophy but did not reveal any evidence of crescent formation. Immunofluorescence revealed granular immunoglobulin M (IgM) and C3 positivity along glomerular capillary basement membranes [2]. The patient was diagnosed with MPO vasculitis and started on prednisone 60 mg daily with an improvement in her shortness of breath and bullous skin lesions. As the patient’s kidneys had already sclerosed more than 70%, she was unable to be weaned off HD.

## Discussion

MPO vasculitis predominantly affects small vessels, commonly involving the kidneys. Renal histopathological findings in MPO-ANCA vasculitis typically lack immune deposits and crescent formation, contrasting with the increased IgM deposits observed in this case [1]. Untreated cases with renal involvement are associated with high mortality rates. Prompt recognition and initiation of corticosteroid therapy are crucial for preventing renal failure and improving patient outcomes.

## Conclusions

This case underscores the significance of recognizing atypical presentations of MPO-ANCA vasculitis, particularly in cases with rapidly declining renal function. Early intervention with corticosteroids is essential in mitigating renal damage and improving prognosis.
